# Aversion Therapy for the Football Hooligan

**Published:** 1990-06

**Authors:** M. G. Wilson


**Aversion Therapy for the Football Hooligan**

*Our object all sublime,*
*We will achieve in time,*
*To make the punishment fit the crime.*

Is the football hooligan a criminal, a psychopath or merely someone exhibiting a familiar, if atavistic, male behaviour pattern? A pattern no doubt encouraged by the loss of will to impose discipline among post-war parents and educationalists. I would argue that he is in the last group. Such people form an element in any sample of humans. Wellington called them the scum of the earth and said he hoped they frightened the enemy as much as they frightened him. He was able to exploit them for the nation's benefit. In the era of empire when there were always wars going on large and small, such people were useful. Nowadays, at any rate in Europe, peace and tranquility are the norm, all instinctive aggression cannot be channelled into sport and the 'martial arts' and it seems we have no remedy except fines and imprisonment. An erstwhile Mikado stated his '*object all sublime*', laid down some sound principles and gave some interesting examples. The chattering society bore is '*sent to hear sermons from mystical Germans*'. The amateur tenor is made to '*exhibit his powers to Madam Tussaud's waxwork*'. The billiard sharp is made to '*play extravagant matches in fitless finger stalls, on a cloth untrue with a twisted cue and elliptical billiard balls*'. These are all examples of aversion therapy. How can we induce aversion in the hooligan, football or other? It should not be too difficult.

A remote area that can be isolated from the public is chosen, perhaps an army firing range, here an arena is surrounded with electrified barbed wire. At the time of the incident, a group of 50–100 warring hooligans is rounded up by the police from the terraces or the streets and driven away to the prepared arena. Half of them are sprayed red and the other half blue and they are segregated into the two halves of the arena. Each man is given a crate of non-alcoholic beer.

After an interval clubs are distributed to all and the barrier removed. They will be told that they are on their own for a couple of days and that they can indulge in their favorite sport without interference, when they are visited again it is hoped they will all have enjoyed themselves and casualty treatment will be available for survivors. Loudspeakers will relay sounds of mass cheering and bloodthirsty exhortations in the vernacular. Periodically showers of empty plastic bottles will descend in random fashion and those watching on the video monitors will be able to drench the arena from time to time if things appear to be getting out of hand. Floodlighting will illuminate the hours of darkness.

Of course there will be no mass carnage, in fact they will probably not have lifted a finger against each other. They will be bored, hungry, wet and disillusioned, and their appetite for orgies of tribal warfare will have evaporated. They can then be given a meal with a wash and brush up, have their clothing dried and sent on their way—wiser and not sadder men.

All we need is a Mikado! But what would the Mikado have done? He would probably have preferred '*something lingering with boiling oil in it*'.

M. G. Wilson

